# UCP1 modulates immune infiltration level and survival outcome in ovarian cancer patients

**DOI:** 10.1186/s13048-022-00951-z

**Published:** 2022-01-28

**Authors:** Jinfa Huang, Guilian Wang, Kedan Liao, Ning Xie, Kaixian Deng

**Affiliations:** Shunde Hospital, Southern Medical University (The First People’s Hospital of Shunde), Foshan, 528308 Guangdong China

**Keywords:** Ovarian cancer, UCP1, Immune infiltration level, Tumorigenesis, TCGA

## Abstract

**Background:**

The uncoupling proteins (UCPs) are critical genes associated with tumorigenesis and chemoresistance. However, little is known about the molecular mechanism of the UCPs in ovarian cancer (OV).

**Material and methods:**

UCPs expression analysis was conducted using Gene Expression Profiling Interactive Analysis (GEPIA), and its potential in clinical prognosis was analyzed using Kaplan- Meier analyses. The influence of UCPs on immune infiltration was analyzed by TIMER. In addition, the correlation between UCPs expression and molecular mechanisms was investigated by TIMER and Cancer Single-cell State Atlas (CancerSEA).

**Results:**

UCP1, UCP2, UCP3 and UCP5 expression levels correlated with a favorable prognosis and tumor progression. Moreover, UCP1 expression correlated to several immune cell markers and regulated tumorigenesis, such as tumor invasion, EMT, metastasis and DNA repair. In addition, UCP1 potentially involved in genes expression of SNAI2, MMP2, BRCA1 and PARP1.

**Conclusions:**

These results implied a critical role of UCP1 in the prognosis and immune infiltration of ovarian cancer. In addition, UCP1 expression participated in regulating multiple oncogenes and tumorigenesis.

**Supplementary Information:**

The online version contains supplementary material available at 10.1186/s13048-022-00951-z.

## Introduction

Ovarian cancer (OV) is one of the most lethal malignant tumors of the female reproductive system, accounting for approximately 295,414 new cancer cases and 184,799 cancer death in 2018 in the United States [1]. Similarly, 52,100 new cases and 22,500 death of ovarian cancer are projected to occur in China in 2015 [2]. Generally, in the early stage, OV have a 5-year survival rate of about 70–80% [3]; however, in the advanced stage (stage III/IV), the 5-year overall survival (OS) rate is lower than 30% [4]. Ovarian cancer is asymptomatic at early stages and early detection is difficult. Therefore, there is an urgent need for more effective genetic and epigenetic molecular markers for the early diagnosis, prognosis evaluation and early intervention of this disease.

It has been proven that metabolic reprogramming, one characteristic of malignancy, is closely related to tumor occurrence, development, metastasis, chemotherapy resistance and recurrence [5]. This metabolic reprogramming supports specific demands for energy and redox maintenance of tumor cells. One effective strategy for tumor suppression is to dissipate mitochondrial oxidative phosphorylation from ATP synthesis through proton leak. Uncoupling proteins (UCPs) belong to the mitochondrial solute carrier superfamily that increases proton leak on the inner mitochondrial membrane in multiple organs and tissues, including heart, skeletal muscle, brown fat and skin [6].

Evidence has indicated that UCPs are involved in various biological processes in carcinogenesis, such as the proliferation, differentiation, apoptosis, chemotherapy resistance [7]. The role of UCPs in carcinogenesis is still in debate. Some researchers argue that forced UCP2 expression confers a survival advantage on tumors [8], while others have shown that UCP2 expression arrests tumor growth, oxidative stress, and angiogenesis [9]. UCP-3 is elevated in renal cell carcinoma and confers hypoxia resistance on renal epithelial cells [10]. However, relationships between UCPs and prognoses in patients with ovarian cancer and their underlying molecular mechanisms remain unknown.

The findings of this study indicated that high expression of UCP1, UCP2, UCP3 and UCP5 were correlated with better OS in OV patients. Also, we observed that high UCP1, UCP2, UCP3 and UCP5 expressions were associated with favorable outcomes in different clinical stages and grades. Further, analysis showed that UCP1, UCP2, UCP3 and UCP5 were reliable biomarkers for predicting therapeutic effect of debulk operation and adjuvant chemotherapy in OV patients. The expression of UCP1 was crucially correlated with immune infiltration, tumor invasion, epithelial-mesenchymal transition (EMT), metastasis and DNA repair. In summary, our results indicated that UCPs, especially UCP1 might be a prognostic biomarker for OV.

## Methods and material

### Transcriptional expression of uncoupling proteins

Comparisons of UCP1, UCP2, UCP3, UCP4 and UCP5 expression between tumor tissues and normal tissues were analyzed were formed with the FPKM of mRNA in the GEPIA database (Gene Expression Profiling Interactive Analysis) (http://gepia.cancer-pku.cn/) [11].

### Prognostic value of UCPs in ovarian cancer patients

The Kaplan-Meier plotter (www.kmplot.com), an online tool for assessing gene expression on the survival of 6 cancers, was used to evaluate the prognostic significance of UCPs mRNA in OV patients. To assess prognostic value of UCPs, 1657 OV patients were divided into two cohorts using auto select best cutoff algorithm on the website (high vs. low expression) [12]. Briefly, the four genes (UCP1, UCP2, UCP3, UCP5; UCP4 is unavailable) were input into the database respectively to plot the survival curves, log-rank *p*-value being calculated on the web page.

### Immune landscape of UCPs in ovarian cancer

Tumor IMmune Estimation Resource (TIMER, https://cistrome.shinyapps.io/timer/) is used for comprehensive analysis of the immune infiltrate correlation of UCPs in OV patients [13]. When we put 5 genes on the website respectively, TIMER calculated the Spearman’s correlations and displayed statistical significance. We evaluated 5 UCP genes with the infiltration level of different immune cells, involving tumor purity, B cells, CD4+ T cells, CD8+ T cells, neutrophils, macrophages and dendritic cells. To consolidate the analysis results, we further used the correlation module in the TIMER platform to analyzed the association of UCPs and marker genes of immune cells.

### Module analysis of single-cell function

CancerSEA (http://biocc.hrbmu.edu.cn/CancerSEA/) [14] is a database intended for investigating distinct functional states at the single-cell level Single-cell sequencing data in the CancerSEA database are derived from the Sequence Read Archive (https://www.ncbi.nlm.nih.gov/sra), the GEO database and the ArrayExpress database (https://www.ebi.ac.uk/arrayexpress/). The database contains data of 41,900 cancer single-cells from 25 cancers and a total of 280 cell groups were defined. There is a total of 14 functional status in the database, including angiogenesis, apoptosis, cell cycle, differentiation, DNA damage, DNA repair, EMT, hypoxia, inflammation, invasion, metastasis, proliferation, quiescence and stemness. Therefore, we used the CancerSEA database to identify the functional state that UCPs family is related to.

### Statistical analysis

One-way ANOVA test and student t test were used to compare the means of UCPs expression in different groups. Kaplan-Meier analysis and the log-rank test were used for the overall survival analysis of OV patients. Spearman’s correlation coefficients were used to assess the correlation between UCPs and marker genes and immune infiltrating cells. *P* < 0.05 was statistically significant.

## Results

### Differentiated UCPs mRNA expressions in OV patients

**Fig. 1:**
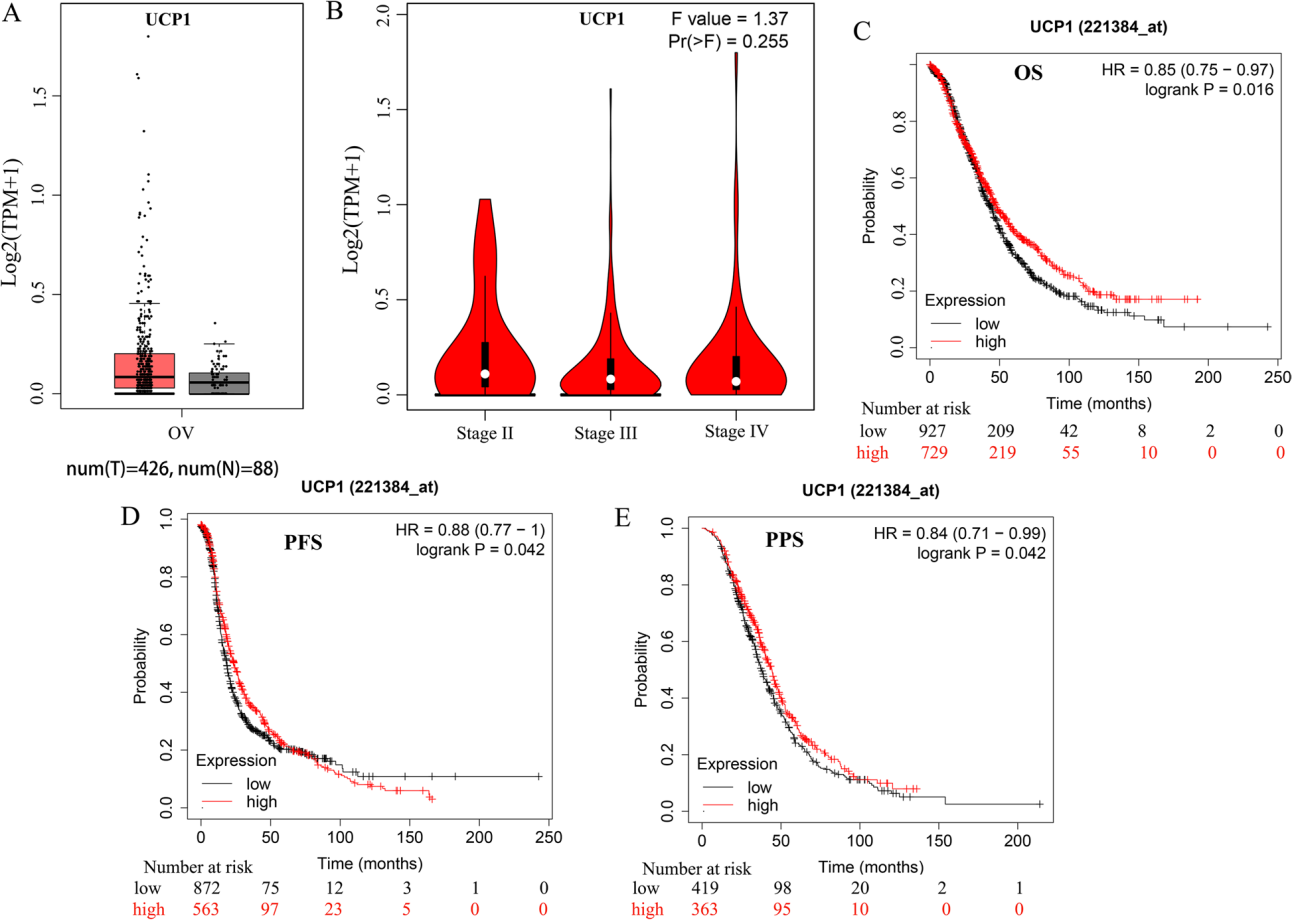
Transcriptional expression level and prognosis value of UCP1 in ovarian cancer. **A** UCP1 transcriptional expression levels in ovarian cancer compared with normal tissues in GEPIA database. **B** UCP1 expression in different stages of ovarian cancer. **C** OS curves of UCP1. High UCP1 expression levels correlated with better OS in OV (*p* = 0.016). **D** PFS curves of UCP1. High UCP1 expression levels correlated with better PFS in OV (*p* = 0.042). **E** PPS curves of UCP1. High UCP1 expression levels correlated with better PPS in OV (*p* = 0.042). (OS: overall survival; RFS: recurrence-free survival; PPS post-progression survival). F-values represent one- way ANOVA

We compared the mRNA levels of UCPs between ovarian cancer and control tissues through the GEPIA (Gene Expression Profiling Interactive Analysis) dataset (http://gepia.cancer-pku.cn/). The results showed that ovarian cancer tissues have a higher expression level of UCP2 than normal tissues (Figure S1A); meanwhile, the expression levels of UCP4 and UCP5 were lower in ovarian cancer tissues (Figure S1I, K). And no differences in UCP1 (Fig. 1A) and UCP3 (Figure S1E) gene expression were observed in tumors when compared to normal tissues. We also analyzed the mRNA levels of UCPs among OV patients with different tumor stages. UCP3 (Figure S1F) and UCP4 (Figure S1J) groups significantly differed, whereas UCP1 (Fig. 1B), UCP2, and UCP5 (Figure S1B, L) groups did not significantly vary.

### Prognostic values of UCPs mRNAs in OV patients

To determine the prognostic value of UCPs, the correlation between the mRNA levels of UCPs and the survival of patients with ovarian cancer was explored by using Kaplan- Meier Plotter tools. Log rank test analyses showed a significant association between the elevated UCP1 (Fig. 1C-E) mRNA levels and improved OS, post-progression survival (PPS), and progression-free survival (PFS) (*p* < 0.05) of all ovarian cancer patients. Ovarian cancer patients with high mRNA levels of UCP2 and UCP3 were predicted to have improved OS and PFS (*p* < 0.05) (Figure S1C-D, G-H). In addition, ovarian cancer patients with higher mRNA levels of the UCP5 (SLC25A14) were predicted to have improved OS and PPS (*p* < 0.05) (Figure S1M-N). Data of UCP4 were not available for ovarian cancer patients.

### Association of UCP mRNA levels with tumor stages and grades of OV patients

**Fig. 2:**
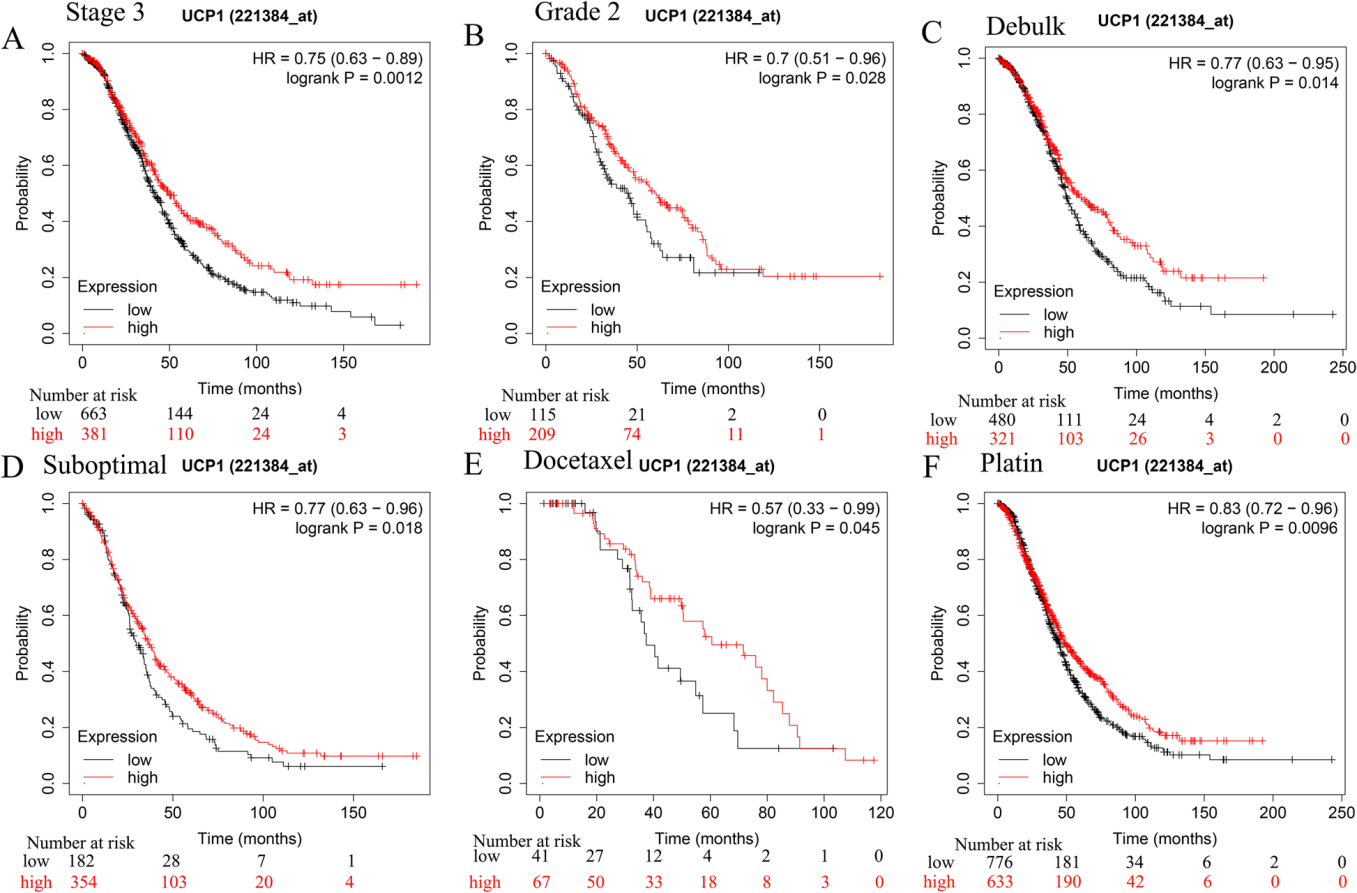
Kaplan-Meier survival curves for ovarian cancer patients according to UCP1 expression levels. **A** and **B** High expression of UCP1 is associated with better survival of patients with stage 3 and grade 2 OV. **C** and **D** High expression of UCP1 is associated with better survival of OV patients with optimal and suboptimal debulk treatment. **E** and **F** High expression of UCP1 is associated with better survival of OV patients with docetaxel and platin chemotherapy

We further explored the correlations between UCPs mRNA levels and the survival of OV patients with different tumor stages and grades using Kaplan-Meier Plotter tools. The results revealed that the increased UCP1 mRNA was significantly associated with improved overall survival of Stage 3 and Grade 3 ovarian cancer patients (*p* < 0.05) (Fig. 2A-B). The elevated UCP2 mRNA expression was associated with improved OS of Stage 1–2 and 3 and Grade 3 ovarian cancer patients (Table S1). In addition, ovarian cancer patients at Stage 3 and Grade 2, 3 were predicted to have longer OS when they have higher mRNA levels of the UCP3 (Table S1). Meanwhile, ovarian cancer patients at Stage 1, 2, 4 and Grade 2, 3 were predicted to have longer OS when they have higher mRNA levels of the UCP5(SLC25A14) (Table S1). The data of UCP4 were not available for ovarian cancer patients.

### Differentiated UCP mRNA expressions in OV patients with different treatments

Surgery and adjuvant chemotherapy are the most common treatments for ovarian cancer. Effectively predicting the prognosis of patients can guide the clinical treatment of cancer patients and avoid waste of medical resources. Therefore, we further analyzed the role of UCPs in the prognosis of ovarian cancer patients after surgery or chemotherapy. Patients with lower mRNA levels of UCP1, 2, 3, and 5 have inferior prognosis after optimal and suboptimal debulk (Fig. 2C-D, Table S2). The most significant prognosis values were shown in UCP3 (HR = 0.69, 95%CI: 0.55–0.86, *p* = 0.00074) for optimal debulk patients and UCP2 (HR = 0.7, 95%CI: 0.57–0.86, *p* = 0.00053) in suboptimal debulk patients (Table S2). Patients with higher mRNA levels of UCP1, 2, 3, and 5 have better prognosis after docetaxel and platinum chemotherapy, with UCP3 showing the largest potential for predicting prognosis of patients with both docetaxel chemotherapy (HR = 0.56, 95%CI: 0.33–0.96, *p* = 0.029) and platinum chemotherapy (HR = 0.76, 95%CI: 0.66–0.89, *p* = 0.00033) (Fig. 2E-F, Table S2).

### Correlations between UCP expressions and immune marker genes

The correlation between UCPs and certain marker genes of immune cells, involving tumor-associated macrophage (TAM), M2 macrophages, T-helper 1 (Th1), regulatory T cells (Tregs) and exhausted T-cells, was analyzed by TIMER. Correlation coefficients greater than 0.2 and less than − 0.2 were considered as meaningful and significant correlation at *p* < 0.05 [15]. Specifically, UCP1 showed significant negative correlation with T cell marker genes CD3E, monocyte marker genes CD86, TAM marker genes CCL2 and CD68; M2 marker genes CD163 VSIG4 (V-set and immunoglobulin domain containing 4) and MS4A4A and Th2 marker genes GATA3, as well as positive correlation with B cell marker genes CD19 (Table 1). However, no significant correlation between the other UCPs and immune cell marker genes.

**Table 1:** Correlation analysis between UCP1 and related genes and markers of immune cells in ovarian cancer

Description	Gene markers	Spearman’s Correlation	*P* value
B cell	CD19	0.25	9.20E-06
T cell	CD3E	−0.207	2.56E-04
Monocyte	CD86	−0.229	4.98E-05
TAM	CCL2	−0.242	1.80E-05
	CD68	−0.224	7.60E-05
M2	CD163	−0.228	5.40E-05
	VSIG4	−0.247	1.21E-05
	MS4A4A	−0.24	2.15E-05
Th2	GATA3	−0.225	7.16E-05

*Th* T helper cell, *TAM* tumor-associated macrophage, both *P* < 0.05 and Spearman’s correction> 0.2 are listed in the table

### Relationship between UCP mRNA levels and tumor functional states

**Fig. 3:**
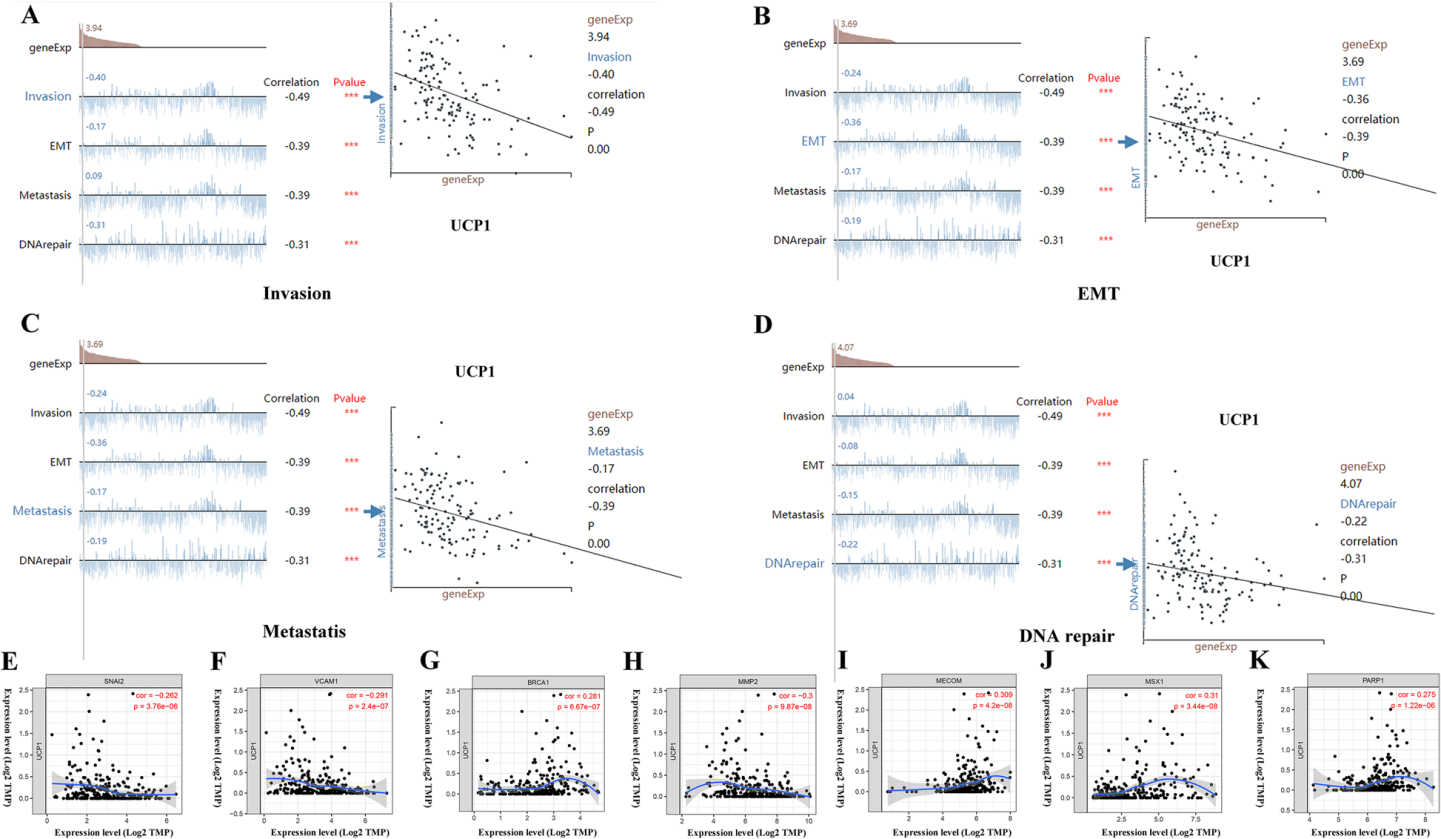
Correlation analysis between UCP1 and functional status of cancer cells. **A** Scatter plot showed the correlation between the UCP1 and invasion. **B** Scatter plot showed the correlation between the UCP1 and EMT. **C** Scatter plot showed the correlation between the UCP1 and metastasis. **D** Scatter plot showed the correlation between the UCP1 and DNA repair. **E** Correlation between UCP1 gene and SNAI2 gene. **F** UCP1 gene and VCAM1 gene. **G** UCP1 gene and BRCA1 gene. **H** UCP1 gene and MMP2 gene. **I** UCP1 gene and MECOM gene. **J** UCP1 gene and MSX1 gene. **K** UCP1 gene and PARP1 gene

Additionally, we analyzed the relevance of UCPs mRNA expression levels to 14 functional states in ovarian cancers, including angiogenesis, apoptosis, cell cycle, differentiation, DNA damage, DNA repair, EMT, hypoxia, inflammation, invasion, metastasis, proliferation, quiescence and stemness, using the CancerSEA database. The results showed significantly strong negative correlations between UCP1 and invasion (*r* = − 0.49, *p* = 0.00), EMT (*r* = − 0.39, *p* = 0.00), metastasis (*r* = − 0.39, *p* = 0.00), DNA repair (*r* = − 0.31, *p* = 0.00) (Fig. 3A-D). At the same time, strong negative correlations between UCP3 and invasion (*r* = − 0.35, *p* = 0.00) (Figure S2A), UCP5 and angiogenesis (*r* = − 0.38, *p* = 0.00) (Figure S2B), were identified. However, UCP2 was correlated with none of 14 functional states.

### Relationship between UCP1 mRNA levels and tumor marker genes

Several genes have been confirmed to be closely related to the occurrence and progression of ovarian cancer, including SNAI2 [16], VCAM1 [17], BRCA1 [18], MMP2 [19], MECOM [20], MXS1 [21], PARP1 [22] and so on. We further analyzed the association between UCP1 and these gene expression. Results showed that UCP1 has negative correlation with SNAI2 (*r* = − 0.262, *p* = 3.76E-06), VCAM1 (*r* = − 0.291, *p* = 2.4E-07) and MMP2 (*r* = − 0.3, *p* = 9.87E-08), while positive correlation with BRCA1 (*r* = 0.281, *p* = 6.67E-07), MECOM (*r* = 0.309, *p* = 4.2E-08), MSX1 (*r* = 0.31, *p* = 3.44E-08) and PARP1 (*r* = 0.275, *p* = 1.22E-06) (Fig. 3E-K). The above evidence indicates that UCP1 is negatively correlated with proto-oncogenes and positively correlated with tumor suppressor genes.

## Discussion

To the best of our knowledge, the present study was the first one to systematically analyze the associations between the expressions of UCPs and prognosis of patients with ovarian cancer. The study showed that expressions of UCP1, UCP2, UCP3 and UCP5 were associated with survival of OC patients. Tumor immune microenvironment analysis indicates that correlations between levels of immune cells’ infiltration and expression of UCPs vary among UCPs family members. Based on cancer single cell sequencing data, tumor functional status analyses suggest that UCP1 may down regulate invasion, EMT, metastasis, DNA repair and angiogenesis.

Recent studies have shown that UCPs are involved in tumor progression, remodeling tumor microenvironment [7], chemoresistance [23] and survival of cancer patients [24]. Given the functional significance of UCPs in cancer, several studies suggested that UCPs might be a reliable marker for predicting prognoses of tumor patients. Through the expression analyses of GEPIA data, we found that compared with the GTEx groups (no TCGA normal data available for OV), OV have higher UCP2 and lower UCP4, UCP5 mRNA levels. Analyses of UCP mRNA levels in different stages of OC showed that UCP3 and UCP4 have distinct expression patterns in different tumor stages, indicating that UCP3 and UCP4 may participate in tumor progression.

UCPs have both cancer-promoting and tumor-suppressing effects on human cancers. Tumor-suppressing role of UCP2 is supported by facts that forced UCP2 expression attenuates cell proliferation in vitro in both mouse and human cancer cell lines. UCP2 deletion contributes to an increased number of colon tumors and a reduced survival rate in mice when exposure to azoxymethane (AOM) followed by addition of dextran sodium sulfate (DSS) treatment [7]. Through survival analyses of the Kaplan- Meier Plotter, we determined clinical value of UCPs in predicting the prognosis of patients with ovarian cancer. The survival analysis revealed that highly expressed UCP1, UCP2, UCP3 and UCP5 were associated with longer OS, PPS and PFS in patients with ovarian cancer. Further, subgroup analysis showed that an increased UCP1 level favored longer OS in patients with ovarian cancer at stage 2 and grade 3, an increased UCP2 level favored longer OS in patients with ovarian cancer at stage 1, 2, 3 and grade 3, an increased UCP3 level favored longer OS in patients with ovarian cancer at stage 3 and grade 2, 3, an increased UCP3 level favored longer OS in patients with ovarian cancer at stage 3, grade 2, 3, and an increased UCP5 favored longer OS in patients with ovarian cancer at stage1, 2, 4, grade 2, 3. Again, highly expressed UCP1, UCP2, UCP3 and UCP5 predicted longer OS in patients receiving both optimal and suboptimal debulk, as well as in patients receiving docetaxel and platinum chemotherapy. These results provided a superficial evidence that UCPs possessed distinct diagnostic potential in OC and the underlying molecular mechanism needed further exploration. Thus, our study highlighted the roles of UCPs in TAM and functional states of OC cells.

Accumulating evidence has shown that immune infiltration is closely related to the proliferation, migration and invasion and chemotherapy resistance of ovarian cancer. Zeng XY et.al found that co-cultured M2-like TAMs with OV cells significantly increased EGF concentration in the supernatants of OV cells and stimulated cell viability, proliferation, migration and invasion which are verified by up-regulated EMT- related markers N-cadherin and Vimentin, and decreased E-cadherin [25]. In our study, we showed negative correlations between UCP1, UCP2 and M2 infiltration, as well as negative associations between UCP1 and M2 marker genes CD163 and VSIG4 in OV. In addition, we also found that UCP3 is positively correlated with both cell infiltration and cell markers of regulatory CD8+ cells, a tumor suppressor cell, indicating that UCP3 may participate in the progression of ovarian cancer by regulating the infiltration of CD8+ cells. Thus, immune infiltration data analysis is consistent with the conclusion of survival analysis.

CancerSEA analysis indicated possible functions of UCPs in OV cells and the results hinted that UCP1 mainly participates in tumor progression by regulating the malignant behavior of ovarian cancer cells, including invasion, EMT, metastasis. At the same time, UCP3 may mediate invasion and UCP5 may participate in angiogenesis in OV. The potential functions of the UCPs were indicative in marker genes. Therefore, it is significant to investigate the association between tumor-associated genes and UCPs. From the results of the tumor-associated genes analysis, the most significant ten tumor- associated genes such as SNAI2, VCAM, MMP2, BRCA1, PARP1 and MECOM were closely associated with UCPs.

Some limitations of this study should be pointed out. First of all, there are many pathological types of ovarian cancer. We analyzed prognosis value of all OV patients without specifying them in different pathological subgroups. Therefore, further study is needed to illustrate whether the predictive value of UCPs is applicable to specific ovarian cancer patients. Secondly, in this study, we did not define the critical value of UCPs expressions. Therefore, in the future, a unified standard should be established to distinguish between high expression and low expression of UCPs in these patients.

## Conclusions

In summary, increased UCP1 expression is associated with better prognosis and increased levels of immune infiltration in ovarian cancer. Moreover, UCP1 participate in regulating multiple oncogenes and tumorigenesis. Therefore, we showed that UCP1 may play an indispensable relevant role in immune cell infiltration levels by regulating oncogenes, and may serve as a potential prognostic biomarker for ovarian cancer. Further in vivo and in vitro studies may help to confirm the function of UCP1.

## Supplementary Information

**Additional file 1 **: **Figure S1**. Transcriptional expression levels and prognosis value of the other UCPs in ovarian cancer. (A), (E), (I) and (K) Transcriptional expression levels of UCP2, UCP3, UCP4 and UCP5 in ovarian cancer compared with normal tissues in GEPIA database. (B), (F), (J) and (L) UCP2, UCP3, UCP4 and UCP5 expression levels in different stages of ovarian cancer. (C) (G) and (M) OS curves of UCP2, UCP3 and UCP5. (D) and (H) PFS curves of UCP2 and UCP3. N PPS curves of UCP5.

**Additional file 2 **: **Figure S2**. Correlation analysis between UCP1 and functional status and markers of cancer cells. (A) Correlation between the UCP3 and invasion. (B) Correlation between the UCP3 and angiogenesis.

**Additional file 3 **: **Table S1**. Association between UCP2, UCP3 and UCP5 levels and patients’ overall survival (OS) in different tumor stages and grades. OR: odds ratio; CI: confidence interval; *P* < 0.05 is statistically significant.

**Additional file 4 **: **Table S2**. Association between UCP2, UCP3 and UCP5 levels and overall survival (OS) of OV patients with surgery and chemotherapy.

## Data Availability

The authors declare that the data and materials of this study are available within the article.
