# Supplementary figures and images for: UCP1 modulates immune infiltration level and survival outcome in ovarian cancer patients
Source: J Ovarian Res. 2022 Jan 28;15:16. doi: 10.1186/s13048-022-00951-z (PMC8800348; doi:10.1186/s13048-022-00951-z)

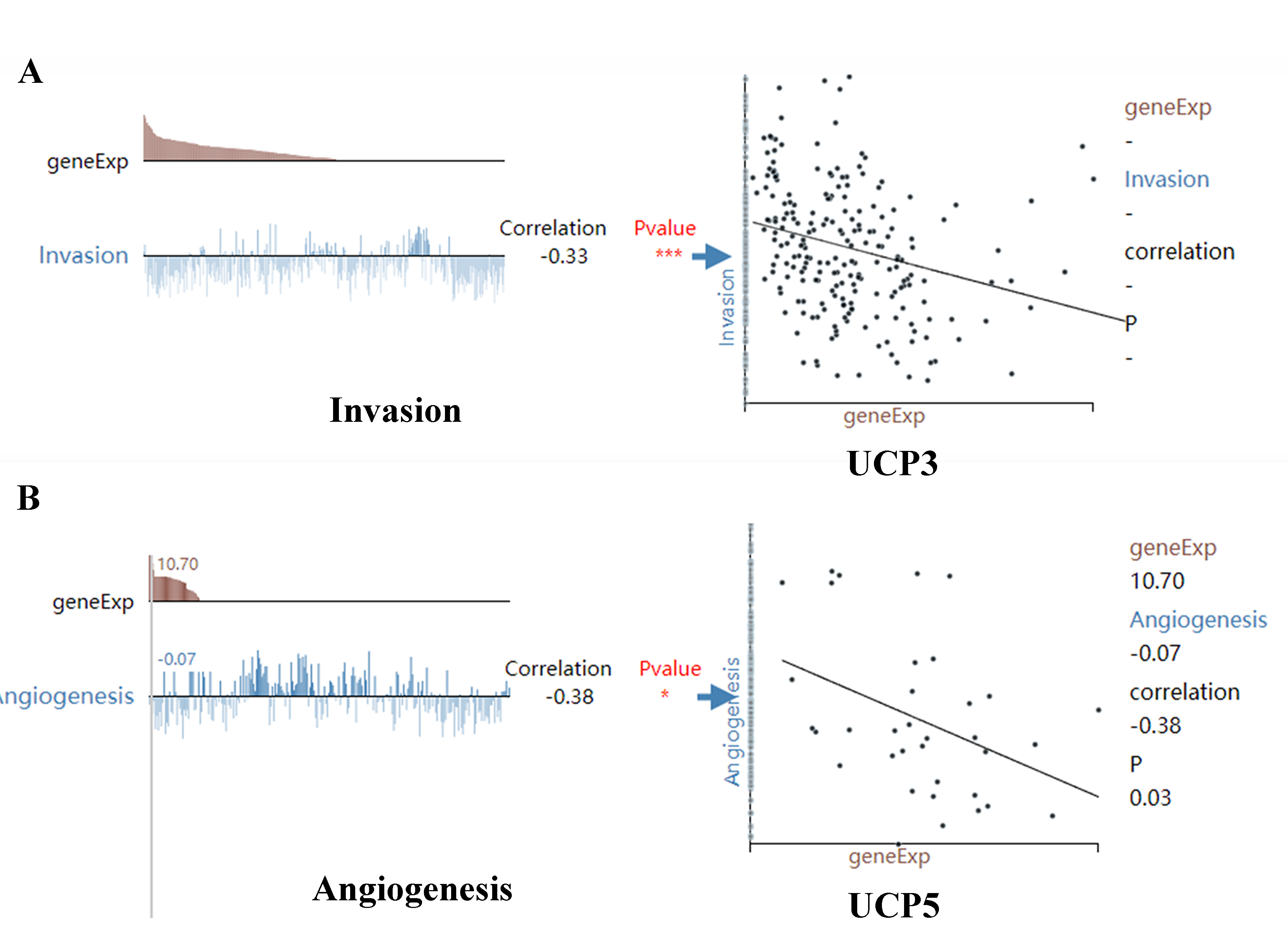

Supplement: Supplementary file 2 — Additional file 2 : Figure S2. Correlation analysis between UCP1 and functional status and markers of cancer cells. (A) Correlation between the UCP3 and invasion. (B) Correlation between the UCP3 and angiogenesis. [file 13048_2022_951_MOESM2_ESM.tif]
