# Supplementary material for: UCP1 modulates immune infiltration level and survival outcome in ovarian cancer patients
Source: J Ovarian Res. 2022 Jan 28;15:16. doi: 10.1186/s13048-022-00951-z (PMC8800348; doi:10.1186/s13048-022-00951-z)
Supplement: Supplementary file 3 — Additional file 3 : Table S1. Association between UCP2, UCP3 and UCP5 levels and patients’ overall survival (OS) in different tumor stages and grades. OR: odds ratio; CI: confidence interval; P < 0.05 is statistically significant. [file 13048_2022_951_MOESM3_ESM.docx]

| Gene | Catagory | OR (95%CI) | P value |
| --- | --- | --- | --- |
| UCP2 | Stage 1 | 0(0-lnf) | 0.017 |
|  | Stage 2 | 0.4(0.2-0.83) | 0.011 |
|  | Stage 3 | 0.78(0.66-0.92) | 0.0032 |
|  | Grade 3 | 0.78(0.66-0.92) | 0.00094 |
| UCP3 | Stage 3 | 0.8(0.67-0.95) | 0.01 |
|  | Grade 2 | 0.68(0.49-0.94) | 0.017 |
|  | Grade 3 | 0.81(0.67-0.98) | 0.029 |
| UCP5 | Stage 1 | 0.2(0.04-0.87) | 0.017 |
|  | Stage 2 | 0.16(0.02-1.21) | 0.042 |
|  | Stage 4 | 0.52(0.36-0.76) | 0.00076 |
|  | Grade 2 | 0.67(0.49-0.92) | 0.014 |
|  | Grade 3 | 0.81(0.69-0.96) | 0.014 |
