# Supplementary material for: UCP1 modulates immune infiltration level and survival outcome in ovarian cancer patients
Source: J Ovarian Res. 2022 Jan 28;15:16. doi: 10.1186/s13048-022-00951-z (PMC8800348; doi:10.1186/s13048-022-00951-z)
Supplement: Supplementary file 4 — Additional file 4 : Table S2. Association between UCP2, UCP3 and UCP5 levels and overall survival (OS) of OV patients with surgery and chemotherapy. [file 13048_2022_951_MOESM4_ESM.docx]

| Gene | | Treatment | OR (95%CI) | P value |
| --- | --- | --- | --- | --- |
| UCP2 | Optimal debulk | 0.79(0.64-0.97) | 0.022 |  |
|  | Suboptimal debulk | 0.7(0.57-0.86) | 0.000 |  |
|  | Platin chemotherapy | 0.79(0.68-0.9) | 0.000 |  |
|  | Taxol chemotherapy  Docetaxel chemotherapy | 0.82(0.68-1)  0.57(0.33-0.99) | 0.049  0.045 |  |
| UCP3 | Optimal debulk | 0.69(0.55-0.86) | 0.000 |  |
|  | Suboptimal debulk | 0.81(0.65-1) | 0.046 |  |
|  | Platin chemotherapy  Docetaxel chemotherapy | 0.76(0.66-0.89)  0.56(0.33-0.95) | 0.000  0.029 |  |
| UCP5 | Optimal debulk | 0.68(0.53-0.87) | 0.002 |  |
|  | Suboptimal debulk | 0.79(0.65-0.97) | 0.024 |  |
|  | Platin chemotherapy  Taxol chemotherapy  Docetaxel chemotherapy | 0.78(0.66-0.92)  0.74(0.62-0.9)  0.5(0.27-0.95) | 0.003  0.002  0.032 |  |
